# Harvesting rice’s dispensable genome

**DOI:** 10.1186/s13059-015-0787-x

**Published:** 2015-10-01

**Authors:** Rod A. Wing

**Affiliations:** University of Arizona, Arizona Genomics Institute, School of Plant Sciences & Department of Ecology and Evolutionary Biology, Tucson, Arizona 85750 USA; International Rice Research Institute, TT Chang Genetic Resources Center, Los Baños, Laguna 4031 Philippines

## Abstract

A rapid and cost-effective approach has been developed to harvest and map the dispensable genome, that is, population-level natural sequence variation within a species that is not present in static genome assemblies.

See related Research article: http://www.genomebiology.com/2015/16/1/187.

## Introduction

Today’s genome assemblies lack crucial information that is necessary to answer many complex functional and evolutionary questions. In a recent publication in *Genome Biology*, Yao et al. [1] described the development and validation of a groundbreaking approach aimed at identifying, mapping, associating genome sequence with agronomic traits/metabolites, and bringing to the forefront previously unknown pan-genome data that can be used to advance biological discovery.

The goal of reference-quality genome-sequencing projects is to provide public access to the majority of a genome, including both gene and repeat space, with high accuracy and contiguity. Reference-quality genomes are long-lasting foundational resources that empower large scientific communities in their efforts to address pressing issues facing humanity, such as disease, food security, and climate change. Unfortunately, the majority of genome assemblies available today are not reference quality and are in need of significant upgrades if they are to achieve this standard.

Even reference-quality sequences are not ‘perfect’ representations of a genome in situ and they are definitely not representative of entire species. For example, the highest-quality genome of any crop plant available to date is that of rice (*Oryza sativa* ssp. *japonica* cv. Nipponbare) published by the International Rice Genome Sequencing Project (IRGSP) 10 years ago [2]. The IRGSP sequenced the rice genome using a bacterial artificial chromosome (BAC)-by-BAC approach, in which each BAC was shotgun sequenced, assembled, finished to an error rate of 1 in 10,000 nucleotides or less, and assembled into 12 pseudomolecules. Over 600 genes have now been cloned from rice, yet many of these are not present in the Nipponbare genome itself, including those for rice grain width or weight (*GW5*) [3], submergence tolerance (*Sub1A*) [4], and resistance to rice blast fungus (*Pikm-1*) [5].

## Capturing the natural variation present in a single species

One approach for capturing more of a species’ genetic variation is to generate multiple high-quality reference sequences (RefSeqs) from accessions that represent various subpopulations and then to use these RefSeqs to assemble a ‘pan-genome’. The pan-genome can then be probed with population re-sequencing data to pinpoint single nucleotide polymorphisms (SNPs) and haplotype and structural variation within the whole species.

A clever and cost-effective alternative, developed by a group from Huazhong Agricultural University in Wuhan China, led by Weibo Xie [1], skips the expensive multi-high-quality RefSeq step and goes directly to the root of the problem. Briefly, Yao et al. [1] used low coverage (1–2.5×) re-sequencing data from 1483 cultivated rice accessions, which they divided into two subpopulations: *indica* (containing the *indica* and *aus* subgroups) and *japonica* (containing the temperate and tropical subgroups of *japonica*). These data were mapped to the IRGSP RefSeq and to three additional *Oryza* assemblies (one Sanger and two Illumina) of varying quality. The reads that did not map to the RefSeq were subsequently assembled. This resulted in an *indica* assembly of ~52 k contigs (N50 = 2344 kb) and a *japonica* assembly of ~30 k contigs (N50 = 2219 kb), henceforth tagged the ‘dispensable genome’, or the non-essential genome, of cultivated rice. These assemblies contain sequences that are not common in all members of the species, or that are unique to one individual. Only ~7700 contigs overlapped between the *indica* and *japonica* assemblies, leading the authors to suggest that the majority of the contig assemblies in each dispensable genome were subpopulation specific.

To validate their contig assemblies, Yao et al. [1] used several approaches, such as PCR amplification with 43 primer pairs from random contigs, generating amplicons of the expected sizes in the corresponding accessions. For example, amplification using an *indica*-specific primer pair resulted in an amplicon from an *indica* accession and no amplification from the *japonica* reference accession.

Annotation of the dispensable genome contig assemblies revealed 6000 *japonica* and 8900 *indica* protein-coding genes, of which 1120 and 1913, respectively, were annotated as ‘high-confidence genes’ on the basis of expression and/or homology. Not surprisingly, ~30 % of each dispensable genome was composed of transposable elements.

Because the dispensable genome assemblies were derived from 1483 accessions, the sequence data were derived from many different haplotypes (that is, groups of SNPs that are linked or inherited together in single accessions). To resolve these haplotypes from the dispensable genome of *indica*, each contig was reassembled locally under more stringent conditions, and 70 % of the reassembled contigs produced between four and seven haplotypes. This result demonstrates that contig-level haplotype information can be harvested from a conglomeration of low-coverage population-level re-sequencing data.

## Mapping the dispensable genome

Amazingly, Yao et al. [1] were able to map about 80 % of the dispensable genome assemblies to approximate locations relative to the IRGSP RefSeq using a combination of hanging paired-end sequences and linkage disequilibrium (LD) mapping. The use of hanging paired-end sequences involves aligning one end of a paired-end sequence to the IRGSP RefSeq, whereas LD mapping used the RiceVarMap derived from the same low-coverage dataset that was used to assemble the rice dispensable genome [6]. To validate the mapping, all genes that have been reported to be absent from the IRGSP RefSeq could be assigned their correct position, except for *Sub1*, which had a repeat that mapped to multiple locations. Interestingly, many of the *indica* and *japonica* dispensable genome contigs mapped uniquely to similar locations (5279 contig pairs within 100 kb of each other) based on SNPs rather than sequence similarity, thereby suggesting the presence of dispensable genome insertion hotspots (*n* = 833).

Because a large percentage of the dispensable genome could be mapped, Yao et al. [1] were able to perform genome-wide association studies (GWAS) for agronomic traits and metabolite profiling. These analyses resulted in the association of about 23 % of the metabolite data and around 42 % of trait data with the dispensable genome. Importantly, the metabolite associations with the dispensable genome SNPs were stronger than those with RefSeq SNPs. The authors conclude by stating that the ‘dispensable genome not only provides additional markers, but also candidate genes’ that are a crucial component of any GWAS or functional genomics analysis.

## Perspective

**Fig. 1:**
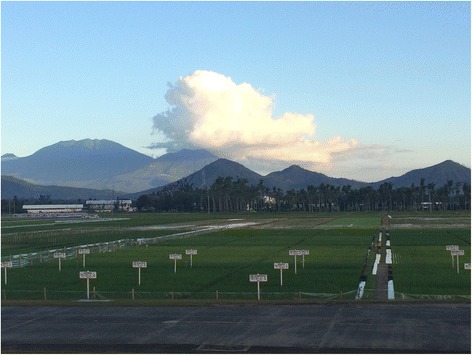
IRRI’s Long-term Continuous Cropping Experiment (LTCCE) is the world's longest-running rice research project. It celebrated its 150^th^ crop last year; that’s three seasons each year for 50 years

Rapid and convenient access to the dispensable genome is crucially important for obtaining a more comprehensive understanding of the natural variation that exists across a species or genus. The dispensable genome contains sequence information that can contribute to growth and productivity under different environmental conditions, and thus can be utilized to help breed the next generation of sustainable and eco-friendly super crops, with the ultimate aim of feeding a global population expected to grow by more than 2.3 billion people by 2050. Rice will play a significant role in helping to solve the ‘9.6 billion people problem’ as it is a major staple crop for around 50 % of the world’s population. Recently, the International Rice Research Institute (IRRI), in collaboration with the Chinese Academy of Sciences and the Beijing Genomics Institute, released re-sequencing data (10–30× coverage) for 3000 rice accessions [7], from among the more than 125,000 accessions archived in IRRI’s TT Chang Genetic Resources Center (Fig. 1). Applying Yao et al.’s. [1] new approach to this data package, in combination with a new set of upgraded and novel reference genomes and high-throughput phenotyping techniques, will empower a giant leap forward in our ability to identify, understand and exploit useful dispensable genome information in order to ensure a safe and stable food supply for generations.

## Concluding remarks

Yao et al. [1] provide compelling evidence that the dispensable genome should not be ignored but rather sought after in any pan-genome study. They described the development and validation of a cost-effective approach to access the dispensable genome of rice, which can be utilized immediately on other plant and animal systems at minimal costs.

As long-read sequencing technology (such as PacBio) matures, high-quality RefSeqs are becoming more affordable. Although it is likely that several new high-quality RefSeqs from single species will rapidly emerge, it is unlikely that it will be possible to capture pan-genome natural variation comprehensively without the utilization of approaches such as that developed by Yao et al. [1].

## Abbreviations

BAC: Bacterial artificial chromosome
GWAS: Genome-wide association studies
IRGSP: International Rice Genome Sequencing Project
IRRI: International Rice Research Institute
LD: Linkage disequilibrium
RefSeqs: Reference sequences
SNP: Single nucleotide polymorphism
